# Sand Erosion Resistance and Failure Mechanism of Polyurethane Film on Helicopter Rotor Blades

**DOI:** 10.3390/polym15224386

**Published:** 2023-11-11

**Authors:** Linfeng Zheng, Jinjuan Fan, Qing Gong, Wei Sun, Xinghui Jia

**Affiliations:** 1 China Helicopter Research and Development Institute, Jingdezhen 333001, China; 288288peak@163.com (L.Z.);; 2 AECC Beijing Institute of Aeronautical Materials, Beijing 100095, China

**Keywords:** polyurethane film, impact angle, sand corrosion resistance, erosion loss mechanism

## Abstract

Polyurethane is widely used on the surface of composite materials for rotor blades as sand erosion protection materials. The failure mechanism investigation of polyurethane film under service conditions is useful for developing the optimal polyurethane film for rotor blades. In this article, the sand erosion test parameters were ascertained according to the service environment of the polyurethane film. The sand erosion resistance and failure mechanism of polyurethane film at different impact angles were analyzed by an infrared thermometer, a Fourier transform infrared spectrometer (FTIR), a differential scanning calorimeter (DSC), a field emission scanning electron microscope (FESEM), and a laser confocal microscope (CLSM). The results show that the direct measurement method of volume loss can better characterize the sand erosion resistance of the polyurethane film compared to traditional mass loss methods, which avoids the influence of sand particles embedded in the polyurethane film. The sand erosion resistance of polyurethane film at low-angle impact is much lower than that at high-angle impact. At an impact rate of 220 m/s, the volume loss after sand erosion for 15 min at the impact angle of 30° is 57.8 mm^3^, while that at the impact angle of 90° is only 2.6 mm^3^. The volume loss prediction equation was established according to the experimental data. During low-angle erosion, the polyurethane film damage is mainly caused by sand cutting, which leads to wrinkling and accumulation of surface materials, a rapid increase in roughness, and the generation of long cracks. The linking of developing cracks would lead to large-scale shedding of polyurethane film. During high-angle erosion, the polyurethane film damage is mainly caused by impact. The connection of small cracks caused by impact leads to the shedding of small pieces of polyurethane, while the change in the roughness of the film is not as significant as that during low-angle erosion. The disordered arrangement of the soft and hard blocks becomes locally ordered under the action of impact and cutting loads. Then, the disordered state is restored after the erosion test finishes. The erosion of sand particles leads to an increase in the temperature of the erosion zone of the polyurethane film, and the maximum temperature rise is 6 °C, which does not result in a significant change in the molecular structure of the polyurethane film. The erosion failure mechanism is cracking caused by sand cutting and impact.

## 1. Introduction

Helicopter rotor blades are mainly manufactured from fiber-reinforced resin matrix composites. During low-altitude–low-speed flight, hovering, or the takeoff and landing of helicopters, sand impacts the surface of the composite blades, causing fiber fracture, the delamination of composites, and the early failure of composite blades [1,2,3,4]. During the Gulf War in the 1990s, the high temperatures and sand dust in Iraq caused severe non-war damage to helicopter rotor blades, and sand erosion became an urgent issue in helicopter design [5,6]. The erosion is mainly caused by sand from two sources: one is the sand in the air, and the other is the sand lifted by the action of rotating rotor blades. From this perspective, the damage of sand erosion for rotor blades is not only related to the natural sand environment but also closely related to the rotation rate of rotor blades [6,7]. Therefore, sand erosion damage on the rotor blades was more severe than that on the general fuselage.

Polyurethane film has a unique micro-phase separation structure containing soft blocks with polymer chain mobility and partially crystalline hard blocks and is, therefore, widely used on the surface of rotor blade composite materials as an anti-sand erosion coating with good impact absorption capacity [6,8,9,10]. The sand erosion resistance of polyurethane film was closely related to the hybrid ratio of the soft and hard segments and the service conditions. The sand erosion failure mechanism of polyurethane film is very complex and depends on multiple factors, which can be divided into internal and external effect factors. The internal effect factors include the properties and structure of the polyurethane film [11,12,13], while the external effect factors include sand particle size, rate, erosion angle, impact flow rate, and other environmental conditions [14,15,16]. It is necessary to understand the sand erosion failure mechanism of polyurethane film under specific service conditions in order to develop the optimal polyurethane film for rotor blades [17,18,19].

In the research of the inner effect factors, Oka [13] studied the effects of polyurethane toughness on impact performance and found that elastic polyurethane materials showed better anti-erosion properties at lower hardness, which was different from that erosion resistance of polyurethane increased with an increase in the hardness [16]. Vishawa and Dong [20,21] found that the addition of graphite oxide, fibers, and carbon black in the polyurethane improved the energy storage modulus and resistance to solid particle erosion. Cho, Wei, et al. showed that the friction coefficient, tensile strength, and fracture strength of polyurethane could affect its impact resistance [18,22].

In the research of the external effect factors, Liu [23] studied the effects of sand erosion rate (50–100 m/s) and impact angle (15–90°) on the erosion wear of polyurethane films on the surface of high-speed trains. The results showed that the higher the impact rate of sand particles, the higher the erosion rate. With an increase in impact angle, the erosion rate increased in the beginning, followed by a decrease, and finally stabilizing. Acierno [24] studied the effect of sand particle impact angle on the erosion rate of polyurethane films at the impact rate of 160 m/s and found that the impact angle with the maximum erosion loss rate was 15°, and when the erosion angle was higher than 45°, the erosion loss rate remained almost unchanged. Dong [25] studied the erosion resistance of polyurethane nanocomposites to solid particles and pointed out that at the impact rate of 10–30 m/s, when the impact angle of solid particles was 30°, the erosion rate of polyurethane nanocomposites was the highest, and at an impact angle of 90°, the erosion rate was the lowest. Doyle [26] studied the erosion behavior of polyurethane under insulation conditions and found that local deformation and high strain led to the temperature increase of the erosion zone, and the mechanical and thermal stresses led to the degradation and loss of the polyurethane. Barkoula, Qi, and Hao et al. [17,27,28,29] believe that there are three main ways in which solid particles impact polyurethane, namely elastic impact, plastic impact, and elastic–plastic impact. Because the polyurethane material has a certain elasticity, pure plastic impact rarely occurs in polyurethane materials. However, in high-angle impact, erosion particles will compress the surface of the material to harden the material, and plastic erosion characteristics will appear in the subsequent erosion process. Fan [30] found that there are a large number of microcracks in the front of polyurethane crack propagation, which absorb the dynamic strain energy generated by impacts.

In the present work, the erosion results cannot evaluate the sand corrosion resistance of polyurethane materials for rotor blades because the failure mechanism of polyurethane coatings is closely related to the experimental conditions. In this article, the sand erosion conditions were determined on the basis of analyzing the rotor blades’ service conditions, and the sand erosion resistance and failure mechanism of polyurethane film were investigated, which could provide technical support for the application and improvement of polyurethane film on rotor blades.

## 2. Specimens and Experimental

### 2.1. Materials and Samples

**Figure 1:**
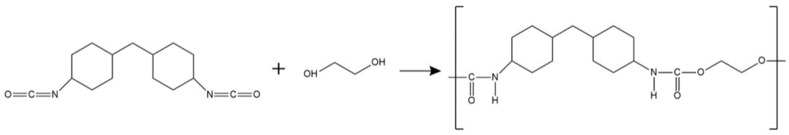
Schematic diagram of polyurethane reaction.

The polyurethane films with a bi-layer structure were fabricated upon composite substrates to give test samples. The surface layer of the bi-layer structure was a polyurethane layer, the thickness of which was 350 ± 10 μm. The bottom layer was a pressure-sensitive adhesive layer (50 ± 10 μm in thickness) used to bond the polyurethane layer to the surface of the composite substrate. The polyurethane layer was an (AB) n-type linear polymer, mainly formed by the reaction of hard segment dicyclohexylmethane diisocyanate (HDMI) and soft segment polyoxypropylene triol (PTMG), as shown in Figure 1. The pressure-sensitive adhesive layer was mainly acrylic ester materials. The polyurethane and pressure-sensitive adhesive are both colorless and transparent materials.

**Figure 2:**
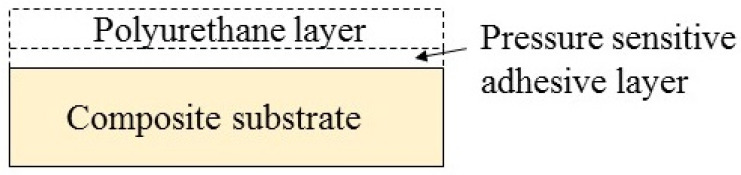
Illustrative cross-sectional image of the sand corrosion specimen.

The composite substrate was a 2 mm thick glass fiber reinforced epoxy resin matrix composite board. The type of epoxy resin was medium temperature epoxy resin, 3238A, which is pale yellow. To fabricate the aforementioned samples, the composite substrate was firstly cut into a 75 mm × 50 mm rectangular plate, the surface of which was then ground with 200 μm sandpaper and cleaned with alcohol. The previously described polyurethane film was cut into a 75 mm × 50 mm rectangle and spread and pasted on the surface of the composite specimen. The polyurethane film was pressed with a roller to prevent bubbles from forming between the pressure-sensitive adhesive layer and the composite substrate, which affected the accuracy of the experimental results. An illustrative cross-sectional image of the sand corrosion specimen is shown in Figure 2. The surface of the prepared sample was light yellow, which was identical to the color of the glass fiber-reinforced resin matrix composite.

### 2.2. Sand Erosion Test

**Figure 3:**
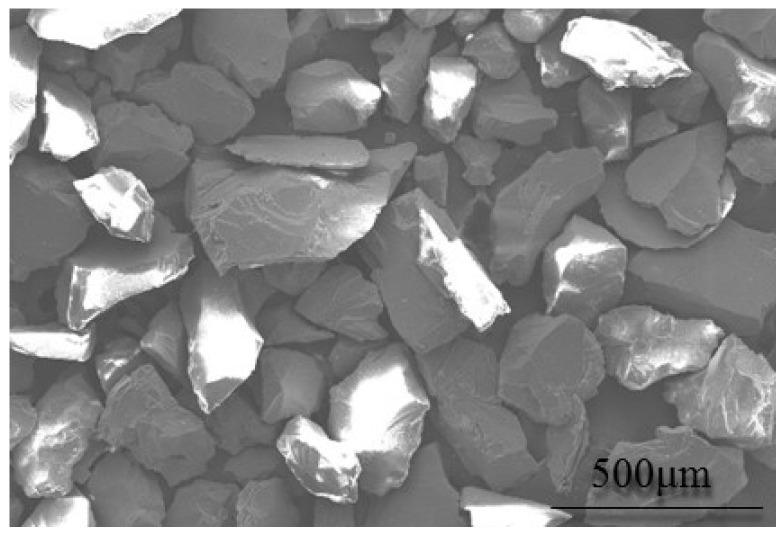
Diagram of the sands.

The sands used in the test are SiO_2_ particles with rhombohedral angles, purchased from the Minghai quartz sand factory, with a size of 80–120 μm, referring to the standard GJB 1171 “Requirements for Sand and Dust Prevention of Military Helicopters”, as shown in Figure 3. The particle size range is measured by AVIC Changcheng Institute of Metrology and Measurement. The results are listed in Table 1.

The sand impact velocity was determined by measuring each condition on actual rotor blades during takeoff and landing, which was equivalent to the helicopter rotor tip speeds and could be calculated by the following Equation [6]:

*v* = 2*πnR* (1)

where

*v*—Sand impact velocity, m/s;

*n*—Blade rotation angle speed, r/s;

*R*—Blade length, m.

In this article, the blade length was 3 m, and the blade rotation angle speed was 11.7 r/s. The sand impact velocity was 220 m/s, calculated by Equation (1).

In general, the serious sand erosion damage was on the leading edge of the blades. The sand impact angles were between 30° and 90° due to the fact that the leading edge of the blade is curved [6]. In this article, the typical impact angles of 30°, 45°, 60°, and 90° were selected to investigate the effect of impact angles on the damage mechanism.

**Figure 4:**
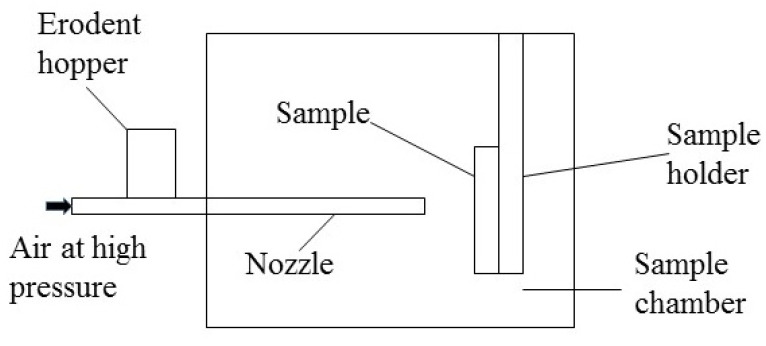
Schematic diagram of sand erosion test device.

**Figure 5:**
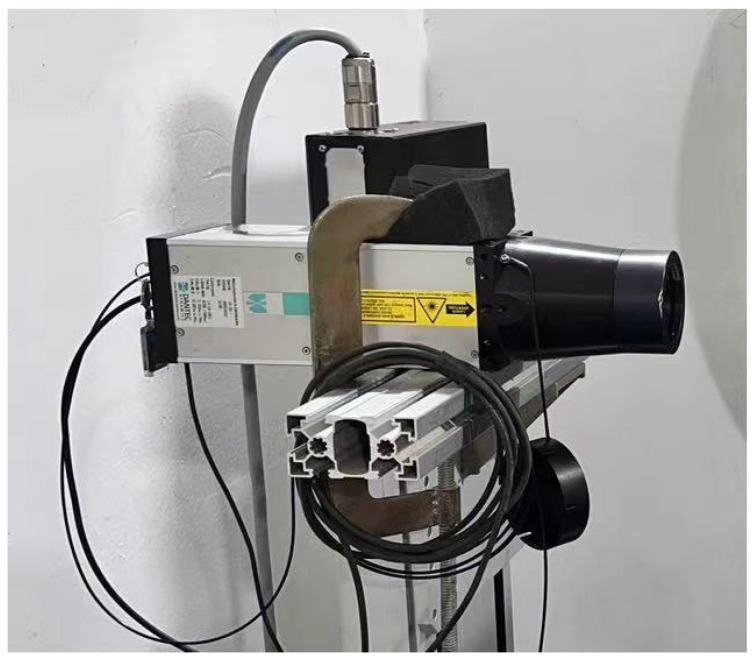
Laser Doppler Velocimetry.

During the experiment, the sand particles were placed in the erodent hopper, then were accelerated in the small diameter nozzle and directed onto the specimen surface illustrated in Figure 4. The impact velocity of sands ejected from the nozzle was controlled by adjusting the pressure of high-pressure air. The particle velocity was measured using a laser Doppler velocimeter, as shown in Figure 5. Firstly, the laser velocity measurement point was adjusted to align with the nozzle of the erosion test device, which made particles pass through the laser. Then, the laser Doppler velocimeter was turned on, and the particle velocity data were collected. The sand velocity was collected three times for each test, and the average velocity value was taken as the final velocity. The test accuracy is ±0.2%. The sand flow was controlled to 6 g/min by adjusting the flowmeter of the hopper. The impact angle was set by adjusting the sample holder. The detailed test conditions are shown in Table 2. The impact time was 2.5 min, 5 min, 10 min, and 15 min respectively. Three samples were tested under each impact angle.

### 2.3. Analysis of Sand Erosion Loss Mechanism

The temperature of the erosion damage zone was measured by an infrared thermometer after the sand erosion test. The accuracy is 0.5 °C. Each damaged area was tested three times, and the average value was taken. The first measurement was taken at the 10th second after the erosion test finished, and the time interval between the two measurements was 5 s. To determine the characteristic functional groups of polyurethane film before and after erosion, the infrared analysis was conducted on the original surface of polyurethane film and the damage zone after sand erosion test by the Spectrum 100 infrared spectrometer. The glass transition temperatures of polyurethane before and after the sand erosion test were analyzed using the DSC25 differential scanning calorimeter. The test conditions were a nitrogen atmosphere, a heating rate of 10 °C/min, and a testing temperature range of −80~300 °C. The microscopic characteristics and the composition change of the sand erosion damage zone were carried out, respectively, using the field emission scanning electron microscope and the energy spectrometer. The volume loss and roughness changes of the damage zone were measured using the OLS4100 laser confocal microscope. During the testing process, the damaged zone was divided into several small zones of 2 mm × 2 mm. The depth and roughness of every small zone were measured and then combined with computer software Zen lite 2012 to obtain the three-dimensional morphology and size of the damage. Referring to standard GB/T1031 [31], statistical Ra with contour height was selected as the roughness characterization parameter. Three samples were measured under each impact angle, and the maximum volume loss and roughness were taken as the final test results.

## 3. Results and Discussion

### 3.1. Erosion Zone Temperature

Doyle’s research [26] showed that local deformation caused by solid particles’ erosion and high-strain insulation conditions led to the temperature increase at the impact zone on the polyurethane. Before the sand erosion test, the samples were placed in the laboratory and adjusted to 25 °C. After the erosion test, the temperature of the damage zone of the samples was tested. Table 3 shows the temperature of the damage zone on the polyurethane film after sand impact at different angles and times.

It can be concluded that the raised temperature in the damage zone gradually decreased with the increase of the sand impact angle at the same impact time, and it increased with the impact time, which is related to the damage degree of the polyurethane, and the more severe the damage, the greater the temperature increase on the damaged zone. When the polyurethane film is impacted for 15 min at the impact angle of 30°, the raised temperature on the erosion damage zone of the sample is the maximum, and the maximum temperature rise was 6 °C. 

### 3.2. Erosion Damage Morphology

**Figure 6:**
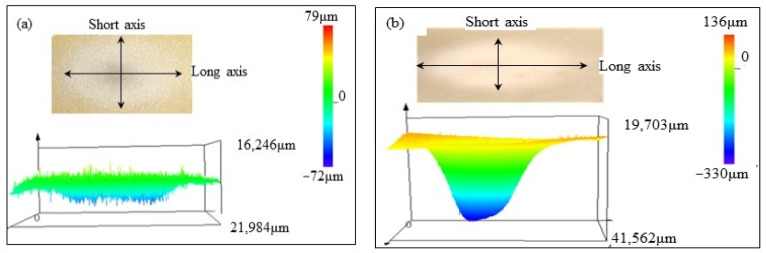
The sand erosion damage morphology impacted for (**a**) 2.5 min and (**b**) 15min at 30°.

**Figure 7:**
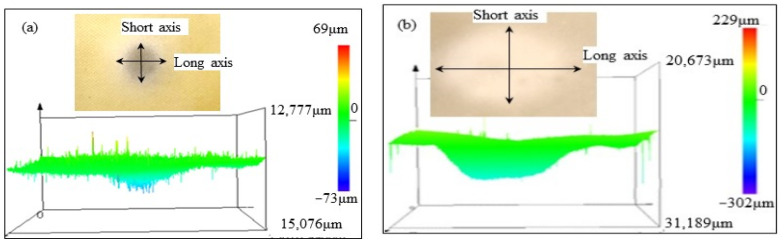
The sand erosion damage morphology impacted for (**a**) 2.5 min and (**b**) 15min at 45°.

**Figure 8:**
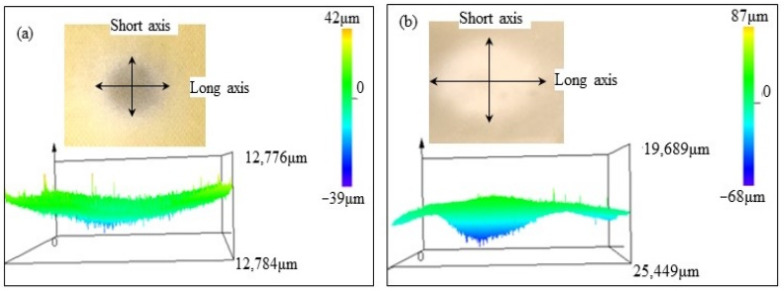
The sand erosion damage morphology impacted for (**a**) 2.5 min and (**b**) 15min at the 60°.

**Figure 9:**
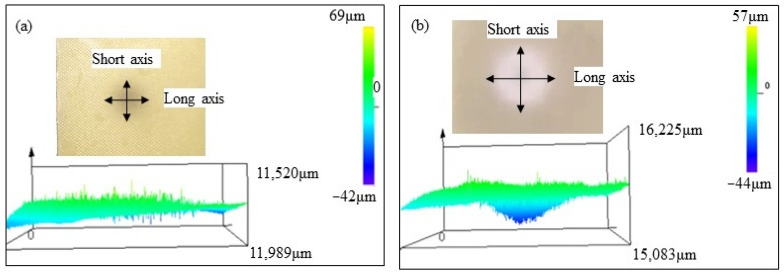
The sand erosion damage morphology impacted for (**a**) 2.5 min and (**b**) 15min at 90°.

The erosion damage morphology and size of polyurethane film at different impact angles are shown in Figure 6, Figure 7, Figure 8 and Figure 9. It can be seen that the morphologies of erosion damage change with impact angles. At the impact angle of 30°, the plane morphology of the erosion damage is approximately elliptical, and the damage area and depth gradually increase with the increasing impact time. When the impact time is 2.5 min, the long axis of the ellipse is 21,984 μm, and the short axis is 16,246 μm. The damage depth is 72 μm. The damage depth profile appears approximately symmetrical. When the impact time is 15 min, the long axis of the ellipse is 41,562 μm, the short axis is 19,703 μm, and the damage depth is 330 μm. The damage depth profile appears to be asymmetric spoon-shaped. Now, the thickness of the remaining polyurethane film is only about 10 μm. 

With the sand impact angle increasing, the ratio of the long axis to the short axis of the ellipse damage morphology gradually decreases. When the sand impact angle is 90°, the sand erosion damage morphology is approximately circular. The area and depth of the damage zone gradually decrease with the increase of the impact angle. The polyurethane film was impacted for 15 min at an angle of 90°; the depth of the erosion damage is 44 μm, which is much lower than that at the impact angle of 30° (330 μm).

The depth profile shapes of the damage zone change with erosion times. When the impact time is short, the depth profile shape of the damage zone is similar to the symmetrical morphology. With the increase in the sand impact time, the depth of damage gradually changed. Except for the sand impact of 90°, the depth of damage zones gradually develops an asymmetric profile. 

### 3.3. Volume Loss

In erosion and wear tests, mass loss is often used to evaluate the material’s resistance to sand erosion [20]. Due to the fact that the polyurethane film used in this article is an elastic material, during the sand erosion test, the sand particles were embedded in the polyurethane film, and the embedded sand mass exceeded the loss mass of polyurethane film, resulting in a significant testing error. Therefore, volume loss is used to evaluate the polyurethane film’s resistance to sand erosion. Moreover, volume loss cannot be calculated through mass loss while obtained by direct measurement methods.

**Figure 10:**
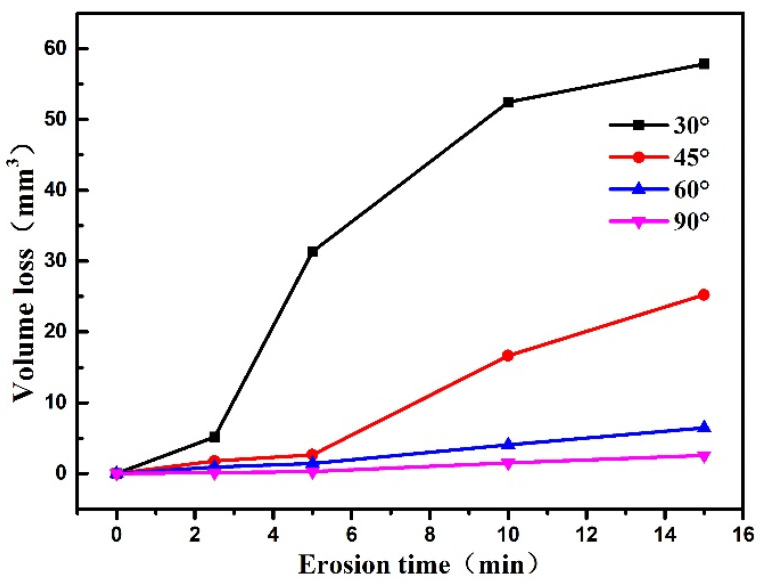
The change of loss volume with sand impact time at different impact angles.

Figure 10 shows the variation of polyurethane film volume loss with erosion time at different erosion angles, where the slope of the straight line represents the rate of volume loss. It can be seen that at different impact angles, with the increase in the sand impact time, the volume loss of the polyurethane film increases. The volume loss rate shows a trend of first increasing and then declining with the impact time. At the same impact time, the maximum volume loss occurs at the impact angle of 30°, and after sand erosion for 15 min, the volume loss is approximately 57.8 mm^3^. With the sand impact angle increasing, the volume loss gradually decreases. When the impact angle is 90°, the volume loss of polyurethane is the lowest. After the sand erosion for 15 min, the volume loss is only 2.6 mm^3^.

Based on volume loss data from different angles, the relationship between volume loss, erosion time, and erosion angle can be expressed as:

(2) $$\begin{array}{c} \;\;V=0.003382t^{4}+0.000512t^{3}\alpha +0.000636t^{2}-0.000196t^{2}\alpha^{2}+0.006145t^{2}\alpha -\\ 0.024131t^{2}-6.812593\times 10^{-6}t\alpha^{3}+0.015535t\alpha^{2}-0.716068t\alpha -0.000715t-\\ 4.191954\cdot 10^{-6}\alpha^{4}-0.138532\alpha^{3}+0.913370\alpha^{2}+18.373836\alpha +6.847228 \end{array}$$

where

V—volume loss, mm^3^;

*t*—erosion time, min;

α—erosion angle, °.

**Figure 11:**
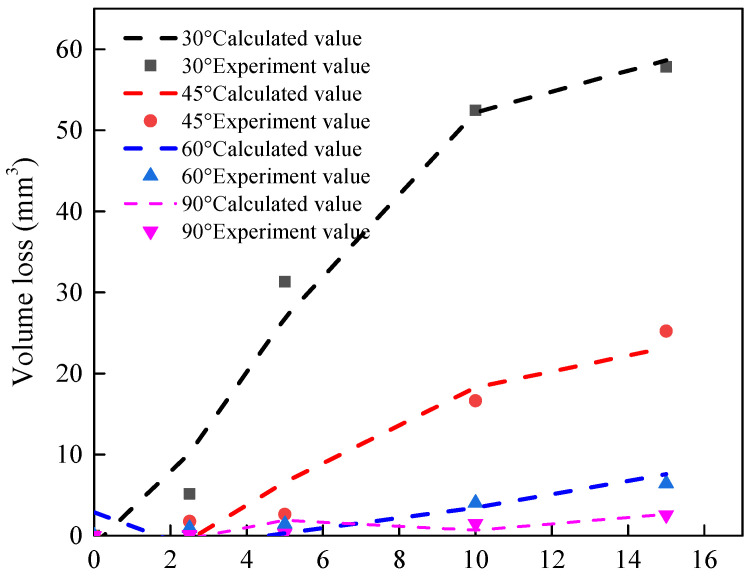
Comparison of experimental and computational results for volume loss.

The comparison of experimental and computational results is shown in Figure 11. The goodness of fit R^2^ = 0.98 shows that the Equation has a good fitting effect.

**Figure 12:**
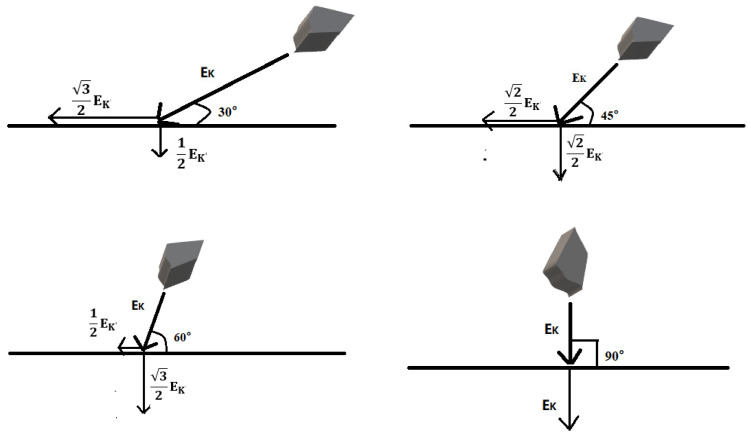
Schematic diagram of erosion load analysis at different impact angles.

When sand particles impact the polyurethane surface at a certain angle, the erosion energy is divided into two components: the component parallel to the erosive surface (tangential energy) and the component perpendicular to the erosive surface (normal energy), as shown in Figure 12. The tangential stress generated by the tangential energy has a cutting effect on the eroded surface, while the normal stress caused by the normal energy impacts the eroded surface. When the sand impact angle changes, the two-component stress also changes accordingly. During the low-angle impact test, the cutting effect of sand is greater than the impact effect, and the damage of erosion particles to the material surface is mainly caused by oblique cutting, while at high-angle impact, the impact effect of sand particles is greater than the cutting effect. The damage of erodent to the polyurethane is mainly caused by direct impact. Based on the damage morphology at different impact angles (from Figure 5, Figure 6, Figure 7 and Figure 8), it can be seen that the damage area and depth of polyurethane film caused by sand cutting are greater than those caused by sand impact.

### 3.4. Surface Roughness

**Figure 13:**
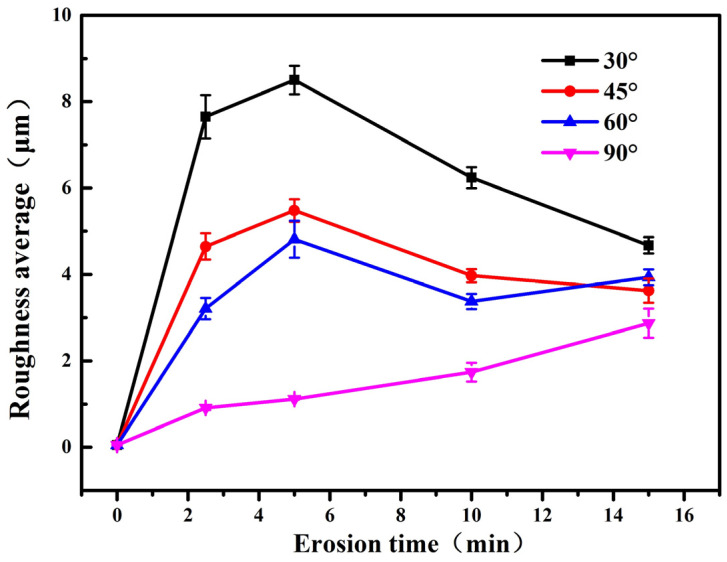
Changes of surface roughness with erosion time at different impact angles.

Figure 13 shows the surface roughness variation of the polyurethane film with the impact time and impact angles. The surface roughness of the fabricated polyurethane film is 0.052 μm. After sand erosions at the impact angles of 30°, 45°, and 60°, the surface roughness of polyurethane first increases and then decreases with the increase in erosion time, while the roughness continuously increases at the impact angle of 90°. At the same impact time, the roughness of the polyurethane surface gradually decreases with the increase in impact angle. The maximum roughness reaches 8.5 μm at the impact angle of 30°.

**Figure 14:**
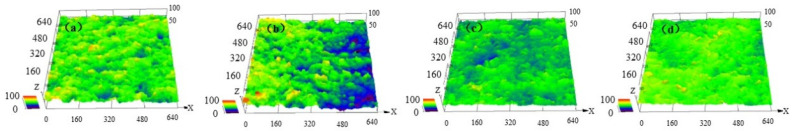
Surface roughness change of polyurethane film at the impact angle of 30° with (**a**) 2.5 min, (**b**) 5 min, (**c**) 10 min, (**d**) 15 min.

During the sand erosion process, when the impact angle is 30°, the shear effect of the tangential load is more likely to cause polyurethane film wrinkling, accumulation, and the formation of peaks and valleys with large height differences on the surface. Therefore, the initial increase rate of roughness is faster. When the surface roughness reaches a certain level, the protruding parts of the surface are easier to be rushed away from the polyurethane surface by the subsequent particle erosion, and a new surface forms. Therefore, there is a slight decrease in the roughness in the later stage of erosion. The variation of surface roughness for polyurethane film with the impact time at the impact angle of 30° is shown in Figure 14. The variation trend of surface roughness at the impact angles of 45° and 60° is similar to that at the impact angle of 30°, and the difference is that the surface roughness changes less. 

**Figure 15:**
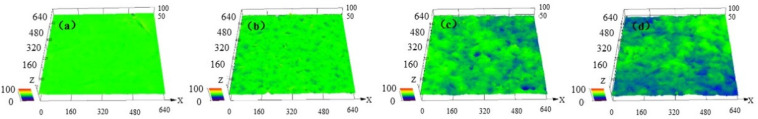
Surface roughness change of polyurethane film at the impact angle of 90° with (**a**) 2.5 min, (**b**) 5 min, (**c**) 10 min, (**d**) 15 min.

At the impact angle of 90°, due to the fact that polyurethane film has good elasticity and some sand particles are directly rebounded, the impact action of erosion particles cannot lead to wrinkling and accumulation of the polyurethane film, and the surface smoothness of the material is maintained well. In the early stage of erosion, the roughness does not change significantly with time. As the erosion time increases, some materials crack and fall off, resulting in an increase in roughness, as shown in Figure 15.

### 3.5. Microscopic Characteristics of Damage

**Figure 16:**
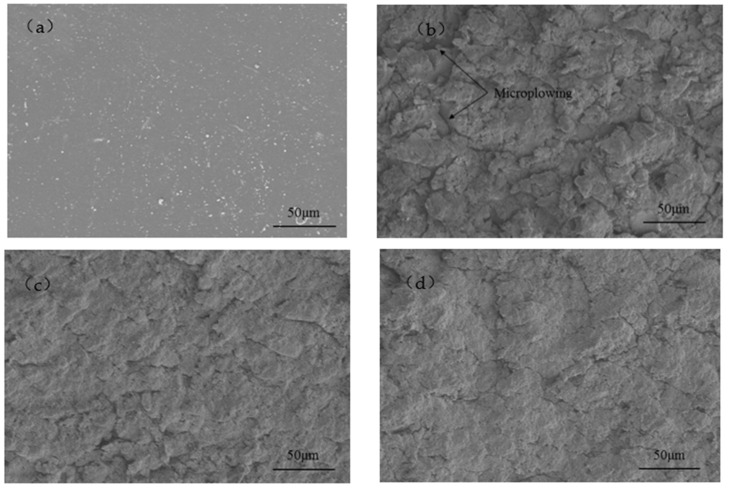

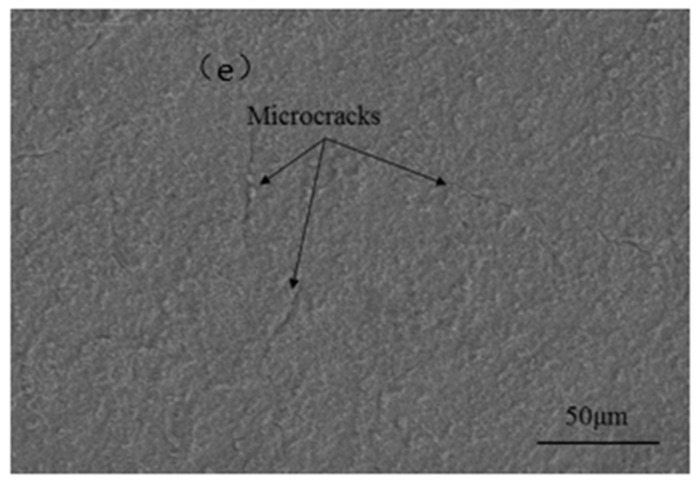
Microscopic morphology of film before and after 15 min of erosion at different impact angles (**a**) as-prepared, (**b**) 30°, (**c**) 45°, (**d**) 60°, (**e**) 90°.

Figure 16 shows the microstructure of the fabricated polyurethane film and 15 min of erosion at the impact angles of 30°, 45°, 60° and 90°. It can be seen that the fabricated coating is flat and smooth, as shown in Figure 15a. After 15 min of erosion at the impact angle of 30°, the polyurethane film surfaces show a plow micro characteristic and become rough. It is mainly micro-cutting marks of sand particles caused by the cut stress parallel to the surface of the polyurethane film. A small amount of microcracks caused by impact stress occurs, as shown in Figure 15b. With the increase in the impact angle, the cutting effect decreases, the plowing marks become shallower, and the cracks on the polyurethane film surface become obvious, as shown in Figure 15c,d. After 15 min of erosion at the impact angle of 90°, the plowing characteristics disappear, and the coating becomes rougher than the original surface. The microcracks caused by impact stress vertical to the film occur, as shown in Figure 15e. It can be concluded that when the polyurethane film is eroded by sand at low angles, the main failure mode is plowing and cutting wear. When it is impacted at high angles by sand, the failure mode is mainly impact cracking. The horizontal and vertical stresses coexist, promoting polyurethane film damage.

### 3.6. Fourier Infrared Analysis

To analyze whether the polyurethane film undergoes chemical changes during the erosion process, infrared analysis was performed on the damage zone after 15 min of 30° erosion because under these conditions, the damage of the polyurethane film is the most serious, and its temperature rise is the maximum.

**Figure 17:**
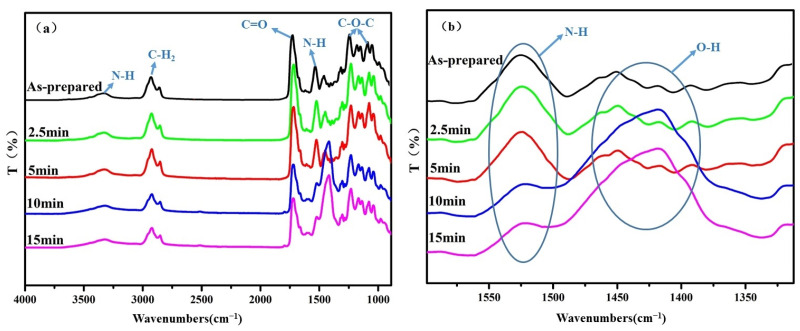
Infrared spectrum of the polyurethane damaged area after 30° erosion at different times with (**a**) full infrared image and (**b**) peak amplification at 1405 cm^−1^.

Figure 17 shows the infrared spectrum of the polyurethane film after sand impact at different times. The peak at 3337 cm^−1^ is the N–H peak, the peak at 2923 cm^−1^ is the asymmetric stretching vibration peak of β-CH2, and the symmetric stretching vibration peak of α-CH2; the peak at 2853 cm^−1^ is the asymmetric stretching vibration peak of α-CH2. The C=O stretching vibration peak of carbamate is at 1717 cm^−1,^ and a CHN deformation vibration peak in the amino ester group is at 1525 cm^−1^. There is a C–O–C stretching vibration peak of carbamate at 1231 cm^−1^, which is a characteristic peak of polyether polyurethane material. The peak at 1078 cm^−1^ is the stretching vibration peak of C–O. It can be seen that after 2.5 min and 5 min of sand erosion, the position and size of the main characteristic peaks of polyurethane film have not changed significantly. After sand erosions for 10 min and 15 min, the characteristic peak of polyurethane film at 1405 cm^−1^ increased significantly, which is the characteristic peak of the adhesive layer pressure-sensitive glue [32]. The depth of the erosion damage is greater than 300 μm after sand erosion for 10 min and 15 min. The remaining polyurethane film is less, and the pressure-sensitive adhesive at the bottom layer has an effect on the infrared radiation. In addition, the C–H–N deformation vibration peak decreases, indicating that there is a small amount of fracture at the connection between the hard and soft segments of polyurethane.

During the erosion test, the temperatures of the polyurethane damage zone rise. As the temperature in the most serious damage zone only increases by 6 °C, it would not cause obvious chemical changes to the polyurethane film. Therefore, the sand erosion of polyurethane film is mainly wear failure.

From the above results, it can be seen that when the depth of sand erosion damage is close to the thickness of the polyurethane film, the infrared spectrum of the damaged polyurethane area changes significantly. Therefore, in the later use stage, whether polyurethane film has reached its service life can be inferred from infrared characteristic peak changes.

### 3.7. DSC Analysis

The glass transition temperatures (Tg) of the soft and hard blocks of the polyurethane film are −21.47 °C and 72.04 °C, respectively. There is no significant change in glass transition temperature after erosion, as shown in Table 4. Furthermore, there was no significant chemical change in the polyurethane film after the erosion test.

### 3.8. Analysis of Sand Erosion Failure Mechanism

In the initial stage of sand erosion, a small amount of sand particles impact the polyurethane film surface, and elastic deformation occurs in the polyurethane film and absorbs some impact energy. With the impact time increasing, the number of impact particles increases, and the material deformation is insufficient to withstand the continuously increasing impact and shear stresses, resulting in plastic deformation, material wrinkling, accumulation of long cracking, and small cracking. The impact time further increased, and the protrusions on the polyurethane surface were sheared and collided into debris removed from the surface, resulting in material loss.

**Figure 18:**
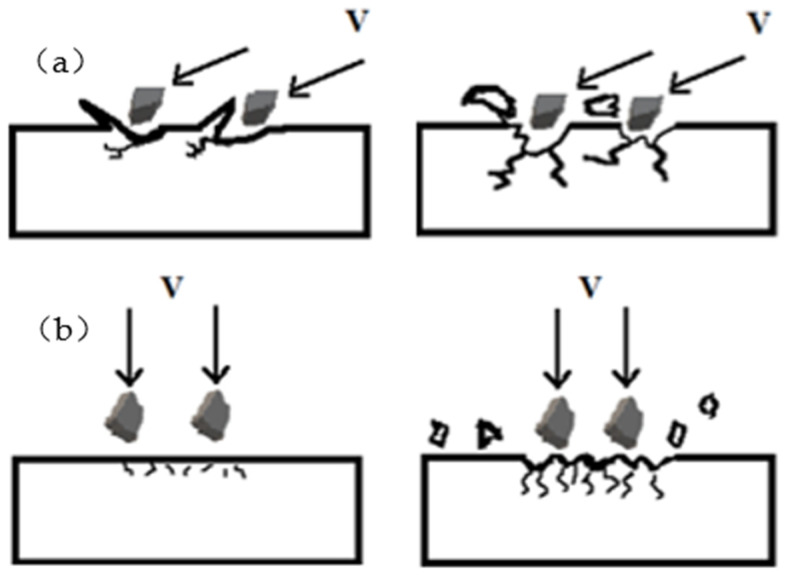
Erosion mechanism of polyurethane film with (**a**) low-angle erosion and (**b**) vertical erosion.

The sand particles with a certain amount of energy impact the surface of polyurethane film, and the erosion failure mechanism changes with the impact angles. During low-angle (30°) erosion, the cutting action of sand particles is greater than the impact action. In the early stage of erosion, the surface of the polyurethane film becomes wrinkled and accumulates under the cutting action of erosion sands, resulting in a rapid increase of surface roughness. With the erosion time increasing, under the action of sand tangential load, some long cracks appear on the surface of the damage zone. The embedding sands in the polyurethane film can also cause cracking of the damage zone. When the cracks propagate and link, some material sheds off from the surface of the film in large pieces, forming a new relatively smooth surface, and the roughness of the damage zone decreases. Under such cyclic erosion action, the material gradually loses and forms asymmetric spoon-shaped damage pits, as shown in Figure 18a.

With the increase of the impact angle, the cutting effect of erosion sands on the polyurethane film decreases while the vertical impact effect increases. During the erosion of vertical (90°) impact, in the initial stage of erosion, material loss is difficult on the surface of the elastic polyurethane film, and the roughness changes little. Then, under the continuous impact of erosive sands, dense and short cracks appear on the surface of the polyurethane film. The propagation and connection of cracks cause the surface material to shed off in small pieces, leading to an increase in surface roughness and ultimately forming symmetrical funnel-shaped erosion damage, as shown in Figure 18b.

As the erosion time increased, the polyurethane film became significantly thinner. After erosion of 10 min, the underlying pressure-sensitive adhesive affected the change of characteristic peak at 1405 cm^−1^. Sand erosion can cause a local temperature rise in the polyurethane film. An increase in temperature may lead to thermal oxygen aging of polyurethane, chemical bond change of molecular chains, and a decrease in physical properties. However, due to the highest temperature only rising to 31 °C, the molecular chain segments had not yet broken or crosslinked, and there was no significant group change in the polyurethane film.

**Figure 19:**
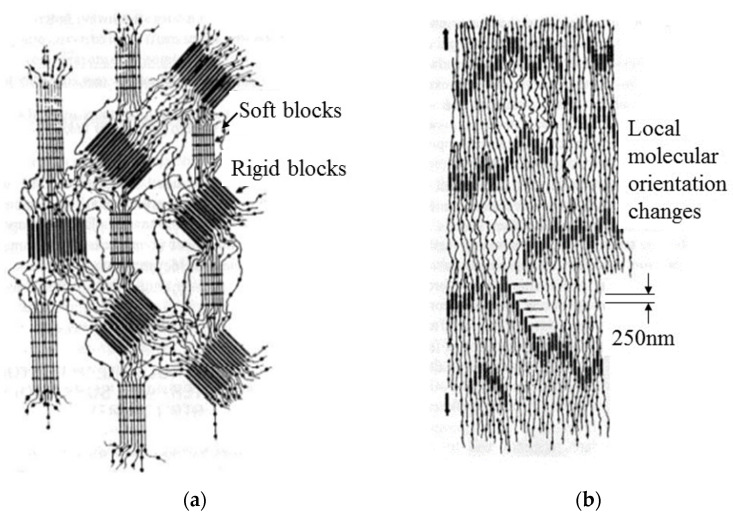
Changes in the arrangement of local soft and hard segments of the polyurethane film during erosion: (**a**) Disordered; (**b**) locally ordered [33].

Figure 19 is a schematic diagram of the polyurethane structure before and after load application [33]. For fabricated polyurethane, the rigid blocks randomly orient and disperse in the flexible block matrix (shown in Figure 19a). These ordered regions are kept in the appropriate positions through intermolecular forces (mainly hydrogen bonds). The local molecular orientation changes due to loading action, and the orderliness of these regions increases (shown in Figure 19b). In this article, the loading is the impact and cutting action of sand. A small amount of fracture occurs in the links between the soft and hard segments with the increase in the erosion time.

After the sand erosion test, under the action of intermolecular forces, the molecular orientation is gradually restored, which is also a manifestation of high elastic deformation. Therefore, during the erosion process, there was no significant chemical change in the polyurethane material. The failure mechanism is cracking caused by sand cutting or impact.

## 4. Conclusions

In this study, the impact velocity of the sand erosion test was determined as 220 m/s according to the service status of polyurethane film on the surface of helicopter rotor blades. The sand erosion resistance and failure mechanism of polyurethane film at different impact angles were analyzed, and the main conclusions are as follows:

The resistance of polyurethane film to low-angle sand erosion is much lower than that to high-angle sand erosion. The erosion damage depth after sand erosion for 15 min at the impact angle of 30° is 330 μm, and the volume loss is 57.8 mm^3^. While the impact angle is 90°, the depth of the erosion damage is only 44 μm, and the volume loss is only 2.6 mm^3^. The direct measurement method of volume loss avoids the influence of sands embedded in the polyurethane film, which can effectively demonstrate the sand erosion resistance of the elastic polyurethane film. The volume loss prediction equation was established. The calculated results are compared with the experimental data, and good agreements are obtained.

During low-angle sand erosion, the failure of polyurethane film is mainly caused by sand cutting. The cutting effect leads to the wrinkling and accumulation of the polyurethane film, a rapid increase in roughness, and the generation of long cracks. The linking of developing cracks leads to the large-scale shedding of polyurethane film. At high-angle sand erosion, the loss of the polyurethane film is mainly caused by impact. The connection of small cracks by impact leads to the shedding of small pieces of polyurethane, while the change in roughness of the film is not as significant as that during low-angle erosion.

During the erosion process of sands, the disordered arrangement of the flexible and rigid blocks for polyurethane film becomes locally ordered under the action of impact and cutting stress. And after the erosion test, the disordered state is restored. The erosion of sand particles leads to an increase in the temperatures of the damage zone, and the maximum temperature rise is 6 °C. At such a temperature rise during a limited testing period, the molecular structure of the polyurethane film does not change significantly. The erosion failure mechanism is cracking caused by sand cutting or impact. In the future, polyurethane film researchers should pay more attention to the shear performance of the material.

## Figures and Tables

**Table 1:** Sand size distribution.

Sand Size (μm)	<40	40~60	60~80	80~100	100~120	120~140	>140
Mass percentage (%)	8 ± 1	12 ± 2	12 ± 3	30 ± 3	23 ± 3	9 ± 2	6 ± 1

**Table 2:** Sand erosion test conditions.

Sand Diameter	Impact Rate	Sand Flow Rate	Impact Angle	Distance from Nozzle to Samples	Nozzle Diameter
80–120 μm	220 m/s	6 g/min	30°, 45° 60°, 90°	100 mm	6000 μm

**Table 3:** The damage zone temperature of polyurethane film (°C).

Impact Angle	Damage Zone Temperature (°C)			
	Impact for 2.5 min	Impact for 5 min	Impact for 10 min	Impact for 15 min
30°	26.7 ± 0.21	28.5 ± 0.20	30.2 ± 0.26	31.0 ± 0.25
45°	26.3 ± 0.20	27.0 ± 0.23	28.3 ± 0.22	30.1 ± 0.25
60°	25.8 ± 0.23	26.5 ± 0.24	28.0 ± 0.20	29.3 ± 0.20
90°	25.5 ± 0.21	26.2 ± 0.20	27.0 ± 0.24	28.0 ± 0.23

**Table 4:** Glass transition temperature Tg (°C) of the damage zone of polyurethane after erosion under different conditions.

Impact Angle	Impact for 2.5 min		Impact for 5 min		Impact for 10 min		Impact for 15 min	
	Soft Blocks	Hard Blocks	Soft Blocks	Hard Blocks	Soft Blocks	Hard Blocks	Soft Blocks	Hard Blocks
30°	−20.73 ± 0.38	74.06 ± 0.49	−19.72 ± 0.44	74.52 ± 0.51	−21.41 ± 0.51	73.17 ± 0.49	−20.01 ± 0.46	72.88 ± 0.49
45°	−20.31 ± 0.46	73.14 ± 0.56	−20.56 ± 0.45	75.20 ± 0.47	−21.06 ± 0.41	73.26 ± 0.57	−22.97 ± 0.57	71.95 ± 0.49
60°	−21.04 ± 0.36	74.04 ± 0.48	−19.56 ± 0.52	73.86 ± 0.42	−20.35 ± 0.48	73.27 ± 0.49	−19.96 ± 0.44	73.18 ± 0.54
90°	−20.21 ± 0.45	72.43 ± 0.64	−21.13 ± 0.48	72.53 ± 0.60	−20.74 ± 0.50	73.17 ± 0.66	−20.83 ± 0.60	73.40 ± 0.47

## Data Availability

The data presented in this study are available on request from the corresponding author.
